# Food and nutrient intake and adherence to dietary recommendations during pregnancy: a Nordic mother–child population-based cohort

**DOI:** 10.29219/fnr.v63.3676

**Published:** 2019-12-20

**Authors:** Carina Madelen Saunders, Eva Maria Rehbinder, Karin C. Lødrup Carlsen, Malén Gudbrandsgard, Kai-Håkon Carlsen, Guttorm Haugen, Gunilla Hedlin, Christine Monceyron Jonassen, Katrine Dønvold Sjøborg, Linn Landrø, Björn Nordlund, Knut Rudi, Håvard O.Skjerven, Cilla Söderhäll, Anne Cathrine Staff, Riyas Vettukattil, Monica Hauger Carlsen

**Affiliations:** 1 Division of Paediatric and Adolescent Medicine, Oslo University Hospital, Oslo, Norway; 2 Faculty of Medicine, Institute of Clinical Medicine, University of Oslo, Oslo, Norway; 3 Department of Dermatology, Oslo University Hospital, Oslo, Norway; 4 Division of Obstetrics and Gynaecology, Oslo University Hospital, Oslo, Norway; 5 Astrid Lindgren Children’s Hospital, Karolinska University Hospital, Stockholm, Sweden; 6 Department of Women’s and Children’s Health, Karolinska Institutet, Stockholm, Sweden; 7 Genetic Unit, Centre for Laboratory Medicine, Østfold Hospital Trust, Kalnes, Norway; 8 Faculty of Chemistry, Biotechnology and Food Science, Norwegian University of Life Sciences, Ås, Norway; 9 Department of Obstetrics and Gynaecology, Østfold Hospital Trust, Kalnes, Norway; 10 Department of Nutrition, Institute of Basic Medical Sciences, University of Oslo, Oslo, Norway

**Keywords:** nutrients, dietary intake, Nordic diet, fetal programming, food intake, nutritional recommendations

## Abstract

**Background:**

A woman’s food intake during pregnancy has important implications not only for herself but also for the future health and well-being of her child. Suboptimal dietary quality has been consistently reported in many high-income countries, reflecting poor adherence to dietary guidelines.

**Objective:**

This study aimed to explore the intake of food and nutrients in a cohort of pregnant women in Norway and their adherence to Nordic Nutrition Recommendations (NNR) and Norwegian food-based guidelines (NFG).

**Design:**

We investigated the dietary intake in 1,674 pregnant women from the mother–child birth cohort, PreventADALL, recruited at approximately 18-week gestational age. Dietary intake was assessed by an electronic validated food frequency questionnaire (PrevFFQ) in the first half of pregnancy.

**Results:**

Total fat intake was within the recommended intake (RI) range in most women; however, the contribution of saturated fatty acids to the total energy intake was above RI in the majority (85.2%) of women. Carbohydrate intake was below RI in 43.9% of the women, and 69.5% exceeded the RI of salt. Intakes of fiber, vegetables, and fish were high in a large part of the population. Many women had a high probability of inadequate intakes of the following key micronutrients during pregnancy: folate (54.4%), iron (49.6%), calcium (36.2%), vitamin D (28.7%), iodine (24.4%), and selenium (41.3%). A total of 22.8% women reported an alcohol intake of >1 g/day, and 4.4% reported an alcohol intake of >10 g/day. Women with higher educational levels showed a tendency towards healthier eating habits, except for higher intakes of alcohol and coffee, compared to women with lower educational level.

**Discussion:**

Excessive saturated fat intake and limited intake of many important micronutrients during pregnancy were common, potentially increasing the risk for adverse pregnancy and birth outcomes.

**Conclusions:**

This study highlights the need for improved nutritional guidance to pregnant women across all educational levels.

## Popular scientific summary

- This population-based study showed that pregnant women had a high intake of fiber, vegetables, and fish.
- Median intake of red meat, salt, and saturated fat was higher than recommended in most pregnancies.
- Over half of the pregnant women likely had a folate intake below the recommended intake.
- Intake of alcohol and coffee exceeded the recommended levels in almost half of the participants, and increased with an increase in the level of education.
- Intervention strategies may be considered to improve maternal nutrition in pregnancy.

A healthy, nutrient-rich, and energy-appropriate diet during pregnancy is crucial for optimal development and growth of the fetus (1). Nutrient requirement is considerably increased during pregnancy and stands in contrast to the recommended modest increase in total energy intake throughout all three trimesters (1, 2). Malnutrition in women and children is a major global health issue, and pregnant women are at increased risk of micro- and macronutrient deficiency (3).

The significance of dietary intake and lifestyle factors during pregnancy has been accentuated with the increasing knowledge of fetal programming on later health outcomes (4–6). Women with a Western diet, characterized by high amounts of saturated fat, sugars, processed foods, and low amounts of fiber (7), during pregnancy are at an increased risk of delivering a child with lower birth weight, whereas adherence to Mediterranean dietary patterns, with high intakes of fruits, vegetables, and fish, has been associated with a decreased risk of delivering infants with lower birth weight (8, 9). Moreover, maternal micro- and macronutrient intake may directly influence the offspring’s organ development, function, and metabolism (10).

In high-income countries, maternal malnutrition tends to manifest itself as a combination of macronutrient overnutrition and micronutrient undernutrition (11). The unfavorable profile of macronutrient intake is commonly reflected by low intake of complex carbohydrates, and high intakes of fat, saturated fat, and salt during pregnancy. Due to the increase in lower-quality diets, inadequate intakes of folate, iron, and vitamin D during pregnancy have consistently been reported in the United States, the United Kingdom, and other European countries (12, 13). Iodine deficiency among pregnant ladies is prevalent in many parts of Europe (14). Although women are advised to abstain from alcohol consumption during pregnancy, about a quarter of women in the general European population reported alcohol use during pregnancy (15).

Nordic countries have for several decades collaborated in developing guidelines for the intake of nutrients, resulting in the Nordic Nutrition Recommendations (NNR) (16). The NNR are intended to support health and prevent diet-associated diseases by setting dietary recommendations for the intake of energy-providing nutrients, intake of micronutrients, as well as fiber and alcohol intake. In 2011, the Norwegian Council for Nutrition published the Norwegian food-based dietary guidelines (NFG), which specify healthy food and lifestyle choices; highlight the need for an increased intake of fruit, vegetables, and whole-grains; and emphasize the importance of limiting the intake of processed and red meat; opting for low-salt foods; and avoiding all forms of alcohol throughout pregnancy (17). The recommended intake (RI) levels for a number of micronutrients are increased in pregnancy. Pregnant women are therefore advised to consume foods with high nutrient density, to ensure adequate intake levels of particularly folate, iron, calcium, vitamin D, and iodine. Evaluation of the diet in a population should thus include both the nutrient- and food-based dietary recommendations.

Assessing dietary intake in large cohort studies is essential for addressing potential diet–disease associations and informing the public about necessary health recommendations (18). Studies reporting on pregnant women’s adherence to dietary guidelines in Nordic countries are few (19, 20). Therefore, the aim of the present study was to explore the intake of food and nutrients, from both diet and supplements, in a recently established cohort of pregnant women recruited from a general population, the Preventing Atopic dermatitis and Allergies in children (PreventADALL) study, and to evaluate their adherence to the NNR and the NFG. In addition, the study aimed to assess coffee and alcohol intake, as well as to evaluate the impact of educational level on dietary intake.

## Study design and methods

### Cohort description

Nutritional data in the present study is derived from the PreventADALL study, a multi-center, prospective, interventional, general population-based mother–child birth cohort study, aimed at the primary prevention of allergic disease. Study design, recruitment, and inclusion criteria are described in detail elsewhere (21).

### Participants and eligibility criteria

Women were recruited from December 2014 to October 2016 by postal invitation from the three participating centers (Oslo University Hospital, Østfold Hospital Trust, and Karolinska Institutet, Stockholm) in connection with their first routine ultrasound examination around gestational week 18. During this period, pregnant women were invited to participate in the study with the following inclusion criteria: singleton and twin pregnancies between weeks 16 and 22 and sufficient Scandinavian language skills. Exclusion criteria were severe maternal or fetal disease. A total of 2,697 pregnant women were enrolled in Norway and Sweden, four of these contributing with two pregnancies.

Study enrollment, baseline interview, and height and weight measures were performed by trained personnel. Weight was recorded to the nearest 100 g by a digital scale, and height to the nearest 1 mm by a stadiometer. Maternal health, pre-pregnancy weight, and sociodemographic and lifestyle factors were obtained through electronic questionnaires, developed in collaboration with the University Center for Information Technology (USIT) at the University of Oslo. The participants received the first general electronic questionnaires within a week after inclusion with an automatic reminder in case of no response. The women were asked to specify the week of gestational age when completing the questionnaires. All women signed a written informed consent prior to study enrollment.

The PreventADALL study was approved by the Regional Committee for Medical and Health Research Ethics in South-Eastern Norway (2014/518) and in Sweden (2014/2242-31/4), and the study was registered at ClinicalTrials.gov (number NCT02449850).

### Dietary assessment

**Fig. 1:**
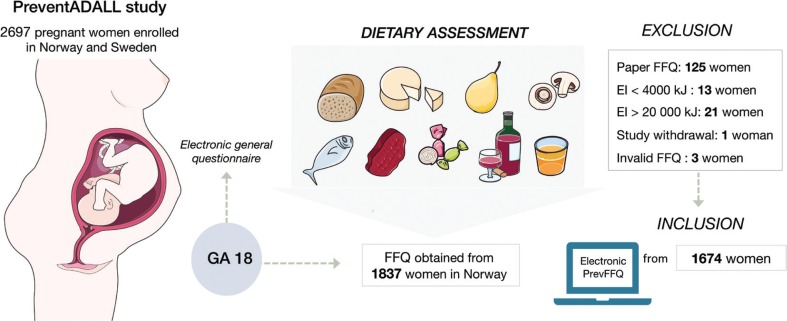
Schematic overview of study and subjects, GA: gestational age, FFQ: Food Frequency Questionnaire. Participants with energy intakes (EI) < 4,000 and > 20,000 kilojoules (kJ) were excluded.

The present dietary assessment was carried out in the Norwegian part of the PreventADALL cohort only, because the dietary assessment method was developed specifically for Norwegian food habits and meal patterns. The pregnant women were given access to the web-based, semi-quantitative food frequency questionnaire (PrevFFQ) via a link sent to their respective e-mails shortly after study inclusion. The PrevFFQ was designed to capture the habitual dietary intake during the first 4–5 months of pregnancy. The FFQ used in the PreventADALL study was previously validated in a population of women, using doubly labeled water and multiple 24-h-recalls (22). The PrevFFQ consisted of 279 questions on the frequency and amount of intake of about 280 food items, grouped according to the main food groups and meal patterns. Frequency categories were used in the increasing order: not at all, times per month, week or day. Amounts were given in portion sizes: standard units, spoons, cups, glasses, etc. The PrevFFQ included pictures of four different portion sizes for food items where portion size may be particularly difficult to estimate. The PrevFFQ included questions about the intake of vitamin or mineral supplements. Omission to answer a question would be followed by an automated comment, prompting the participant to submit an answer. All questions about diet in the FFQ were mandatory, assuring a complete set of values in all questionnaires. The data in the FFQ were transferred to the food and nutrient calculation system, Kostberegningssystemet (KBS), version 7.3, at the Department of Nutrition, University of Oslo, where all estimations of food and nutrient intakes were performed in KBS food composition database AE18. We included only women who answered the electronic questionnaires, excluding the first 125 enrolled women who received a paper version of the FFQ, thereby eliminating potential differences in results due to discrepancies in methods (paper vs. electronic FFQ). The possibility of double participation by four women who were enrolled with two pregnancies was eliminated, as the questionnaire from the first pregnancy was paper-based and therefore excluded. Women who reported unlikely energy intakes (<4,000 kJ/day and >20,000 kJ/day) were also excluded, as well as three invalid FFQs and one study withdrawal, giving a total of 1,674 eligible study participants (23). A study overview and selection criteria are presented in Figure 1. A total of 154 out of the 1,674 women did not answer the 18-week general questionnaire. Therefore, some background information was only available for 1,520 women.

The NNR 2012 defined RI as ‘the amount of a nutrient that meets the known requirement and maintains good nutritional status among practically all healthy individuals in a particular life stage or gender group’ (16). Hence, we present the proportion of women that had intakes within the recommended intake range (RIR) or above the RI, ensuring minimal probability for inadequacy. We did not assess the proportion of the group with relatively high probability of inadequate intake, defined as the proportion below average requirement (AR), as AR levels are not defined for pregnancy in the NNR. We included supplement intake when assessing the micronutrient intake through frequency estimations to increase the reliability of the results (24).

### Statistical analyses

Data were exported from the KBS database and imported to SPSS (Statistical Package for the Social Sciences). All the analyses were performed using IBM© SPSS© statistics version 25 (Chicago, IL, USA). We conducted descriptive analyses of food intake, as well as energy intake, and macro- and micronutrient intake. Normal distribution of variables was investigated through visual inspection of histograms and *p*-plots and by using the Kolmogorov–Smirnov test. Variables are described as means and standard deviations (SD) or median values with interquartile range (IQR) and 5th and 95th percentiles of proportions for normally and non-normally distributed variables, respectively. Descriptive statistics were used to investigate adherence to the recommendations on the frequency of consumption of micro- and macronutrients. Differences between categorical variables were analyzed by Chi-Square test and numerical data by One-Way ANOVA tests. One-way between groups ANOVA was performed to test whether educational level would influence the intake of micro- and macronutrients and food groups. The assumption of normality and homogeneity of variances was tested for each variable.

## Results

Mean (SD) age of the population was 32.5 (4.1) years, and the mean (SD) pre-pregnancy body mass index (BMI) was 24.6 (3.5) kg/m^2^. Other baseline characteristics are presented in Supplementary Table 1 for 1,520/1,674 respondents. Most (90.8%) women had a Scandinavian background, 37.1% had one or more previous deliveries, 61.1% had a university-level education of more than 4 years, 81.0% reported a full-time job, 97.1% were married or cohabitating, and 52.7% had a yearly household income of >1,000,000 NOK.

**Table 1:** Estimated daily intake of macronutrients, salt, and selected foods, compared to dietary recommendations (N = 1,674)

Macronutrient	Unit	Median	Interquartile range	5th Percentile	95th Percentile	Recommended intake range (RIR) (Nordic Nutrition Recommendations 2012 [NNR 12])	Below RIR (%)	Above RIR (%)	Within RIR (%)
**Carbohydrates**	E%	45.7	42.3–49.2	35.9	54.7	45–60 E%	43.9	0.5	55.6
**Protein**	E%	16.5	15.1–18.1	13.1	20.5	10–20 E%	0.2	6.9	92.9
**Total fat**	E%	34.5	31.2–37.8	26.3	43.6	25–40 E%	2.9	14.0	83.1
**Saturated**	E%	12.5	10.9–14.1	8.7	17.0	max 10 E%	14.8	85.2	14.8
**Monosaturated fat**	E%	12.7	11.2–14.3	9.3	17.3	10–20 E%	9.4	0.8	89.8
**Polyunsaturated fat**	E%	5.7	5.0–6.8	4.0	9.2	5–10 E%	23.7	3.3	73.0
**Fiber**	g/day	32.2	25.0–41.3	16.8	55.5	min 25–35	22.4	41.6	36.0
**Food group**						**Recommended intake range (RIR) (Norwegian Food Based Dietary Guidelines [NFG] 2011)**	**Adherence to NFG (%)**		
**Fruit and berries**	g/day	221	141–328	61	623	min 250 g/day	42.1		
**Vegetables**	g/day	363	253–514	141	802	min 250 g/day	75.6		
**Fish + seafood**	g/week	539	188–791	121	1,274	300–450 g/week^a^	19.5 (60.3% >RIR)		
**Fatty fish**	g/week	136	71–206	7	404	min 200 g/week^b^	27.7		
**Red meat**	g/week	516	339–723	116	1,100	max 500 g/week^a^	47.8		
**Salt**	g/day	7.2	5.7–8.9	3.9	12.2	Max 6 g/day	30.5		
**Coffee**	g/day	203	21–405	0	878	170–340 g/day^c^	59.1		
**Alcohol**	g/day	0.1	0.0–0.8	0.0	9.3	0 g/day	56.5		

NNR12 (Nordic Nutrition Recommendations 2012), the NFG (Norwegian Food Based Dietary Guidelines) refer to only Norway; E%: percentage of total energy intake per day;

a Recommendations given in gram per week.

b Included in the total amount of fish and seafood per week.

c Recommendations based on reference values established by the Department of Nutrition (25). Recommended daily intake is 1–2 cups/day. One cup is equivalent to 170 g coffee. Caffeine content was calculated based on European Food Safety Authority (EFSA) guidelines (26): 44.5 mg caffeine/100 g black coffee.

### Intakes and adherence to recommendations for macronutrients and the main food groups

The participants’ intake of macronutrients and selected foods and beverages per day, as well as the proportion of participants with intakes in line with the recommendations, are presented in Table 1.

Median (IQR) energy intake was 10,082 (4,139) kJ. Total fat intake was above RIR (25–40 E%) in 14.0% of the women, and saturated fatty acid intake was above RI (max 10 E%) in 85.2% of the women. Average intake of fats is presented in Supplementary Table 2. Protein RIR (10–20 E%) was met by 92.9%, carbohydrate RIR (45–60 E%) by 55.6%, and fiber RI (> 25 g/day) by 77.6% of the women.

**Table 2:** Estimated daily intakes of selected micronutrients, compared to Nordic Nutrition Recommendations (NNR) (*N* = 1,674)

Variable	Unit	Median	Interquartile range	5th Percentile	95th Percentile	Recommended intake (NNR)	Percentage with intakes	
							Below RI	Above RI
Vitamin A	RE^a^	1,694	1,101	661	3,313	800 RE^a^/day	9.6	90.4
Vitamin C	mg	207	136	86	425	85 mg/day	4.4	95.6
Vitamin D	μg	13.6	12.1	4.2	32.6	10 μg/day	28.7	71.3
Vitamin B 12	μg	7.9	4.1	4.0	14.5	2 μg/day	0.3	99.7
Iodine	μg	256	189	103	563	175 μg/day	24.4	75.6
Folate	μg	480	275	236	921	500 μg/day	54.4	45.6
Zinc	mg^b^	15.5	14.8	7.7	43.4	9 mg/day	10.2	89.8
Calcium	mg	1,045	558	495	1,960	900 mg/day	36.2	63.8
Selenium	μg	69	52	31	162	60 μg/day	41.3	58.7
Iron	mg^c^	15.1	10.0	7.3	92.0	>15^d^ mg/day	49.6	50.4

RI: Recommended intake (NNR 2012) in pregnancy.

a Retinol equivalents: 1 retinol equivalent (re) = 1 μg retinol = 12 μg β-carotene. α-tocopherol equivalents: 1 α-tocopherol equivalent (α-te) = 1 mg rrr α-tocopherol.

b The utilization of zinc is negatively influenced by phytic acid and positively influenced by animal protein. The recommended intakes are valid for a mixed animal/vegetable diet. For vegetarian cereal-based diets, a 25–30% higher intake is recommended.

c Meal composition influences the utilization of dietary iron. Availability increases if the diet contains abundant amounts of vitamin C and meat or fish daily, and it is decreased with simultaneous intake of polyphenols or phytic acid.

d Increased need of iron intake during second and third trimesters in pregnancy.

The recommendations for vegetable intake (>250 g/day) was met by 75.6% and fresh fruit intake (>250 g/day) by 42.1% of women. A total of 52.2% exceeded the recommended intake for red meat (max 500 g/week), and 60.3% exceeded the recommended intake of fish and seafood (300–450 g/week). Daily salt intake was above recommendations (6 g/day) in 69.5% of women. The average intakes of other food groups and beverages are included in Supplementary Table 3.

#### Adherence to recommendations for micronutrients

Table 2 shows the participant’s estimated average daily intake of micronutrients compared to the RI levels (NNR 2012). Micronutrients deemed especially important during pregnancy and the intake of other micronutrients are listed in Supplementary Table 4. An intake below RI was seen in a high percentage of women for the following micronutrients: 54.4% for folate (500 μg/day), 49.6% for iron (15 mg/day), 36.2% for calcium (900 mg/day), 24.4% for iodine (175 μg/day), 41.3% for selenium (60 μg/day), and 28.7% for vitamin D (10 μg/day).

#### Alcohol and coffee

Almost a quarter (22.8%) of the women reported an alcohol intake of >1 g/day, 4.4% an intake of >10 g/day, and 1% an intake of >15 g/day. Women reported their coffee consumption in cups. One cup was defined as 170 ml, equal to 170 g of coffee. Median coffee intake was 203 g/day, and 40.9% of the women reported a daily coffee consumption above the recommended 340 g (27). Median caffeine intake from coffee was 90 mg/day, and 19.4% exceeded a daily intake of 200 mg of caffeine (RI max 200 g/day).

#### Education level

Post hoc comparisons using the Tukey’s HSD test indicated that the median intake of red meat was significantly higher in participants with high school degree only and those with university education ≤ 4 years, compared to women with university education > 4 years (*P* = 0.015). We found a significantly higher intake of fiber (*P* = 0.002), vegetables (*P* = 0.001), and omega-3 (*P* = 0.001) in women with university education > 4 years compared to women with high-school degree only. Coffee consumption increased with an increase in the educational level and was significantly higher in women with a university degree > 4 years compared to high school only (*P* = 0.005). Alcohol intake differed significantly between different educational groups (*P* = 0.004, Kruskal–Wallis test). Women with a university education > 4 years had a significantly higher alcohol intake (*P* = 0.016, adjusted with Bonferroni) compared to those with lower educational level. We found no associations between educational levels and micronutrient intakes. Income levels did not significantly associate with dietary or alcohol and coffee intake.

## Discussion

Our assessment of dietary intake during pregnancy in a mother–child cohort from the general Norwegian population showed that a large number of participants had satisfactory intakes of healthy food items, such as fiber, vegetables, and fish. However, a significant part of our population was at risk for macro- and micronutrient inadequacy, with particularly low adherence levels for folate, iron, selenium, calcium, vitamin D, and iodine. Self-reported red meat, salt, and saturated fatty acid intake exceeded recommendations in more than half of the subjects in the cohort, and together with alcohol and coffee consumption, they were associated with higher educational level.

### Macronutrient- and food intakes

The satisfactory intake of healthy food items by most women in our study is in line with a previous study showing that Nordic diets are commonly characterized by a high consumption of milk and dairy products, moderate to high consumption of meat, and moderate consumption of fruit and vegetables (16). Appropriate birthweight (weight between the 10th and the 90th percentiles) may be facilitated by a maternal plant-food-based dietary pattern, with high intakes of fruit and vegetables, low-fat dairy, and lean meats throughout pregnancy (28, 29). The high vegetable and fiber intake in our cohort, in line with a previous study showing that Norwegian women tend to increase their fruit and vegetable consumption from pre-pregnancy to early pregnancy (30), will likely have positive health effects for both mother and child. A higher intake of fruits and vegetables is also associated with increased infant growth up to 6 months of age (31). Our study was not designed to assess pre-pregnancy dietary habits, but a heightened motivation in pregnancy might have played a role in increasing vegetable consumption in our study population, resulting in the reported high intakes of vegetables. Although estimated vegetable and fiber intake was satisfactory at the group level, more than half of the women in our study population did not meet the recommended daily intake of fruit (250 g/d). Total fish and seafood intake in our cohort was higher than in a study with 119 pregnant Norwegian women reporting a total fish intake of 39 g/day, which is only half of the intake found in our study (32). The high intake of fish and seafood is likely to be beneficial also for the offspring, as maternal fish intake in pregnancy has been associated with positive fetal neurodevelopmental outcomes and reduced levels of allergic disease (33, 34). In spite of its nutritional benefits, fish is also a known source of mercury and other environmental toxins, which can have a negative impact on fetal development (35). Therefore, the benefits of fish intake above the recommended intake levels, as seen in many women in our study, need to be weighed against the potential detrimental effects. The high intake of red meat, salt, and saturated fat in our study is consistent with data from a large meta-analysis concluding that the overall fat and saturated fat intake is above the recommended levels in most pregnant women in high-income countries, whereas carbohydrate intake is below the recommended levels(12). The majority of women in our study had salt intakes above RI levels, which is in line with a Canadian study comprising 1,533 pregnant women reporting sodium intakes above the recommended levels in 85% of the participants (36). Excessive consumption of saturated fat and low intakes of omega 3 fatty acids have been linked to adverse health outcomes in both mother and child (37, 38). Knudsen et al. showed in a large cohort of pregnant Danish women that high intakes of red- and processed meat and high fat dairy was associated with an increased risk of having a child small for gestational age (9).

### Micronutrient intake

Our findings of low adherence to RI for some micronutrients is in line with the consistently reported rise of lower-quality diets in many industrialized countries, leading to an inadequate intake of particularly iron, folic acid, calcium, and vitamin D among pregnant women (39, 40). The potentially inadequate intake of vitamin D, folate, and iron is supported by similar findings in 118 pregnant Finnish women (41). Significant folate deficiencies were observed in about half of the population of 204 Italian pregnant women and women of childbearing age (42). A low reported folate intake, such as observed in our study, is in line with inadequate periconceptional use of folic acid supplement in Oslo (43) and could potentially have serious consequences for the developing fetus (44). Inadequate folate intakes in pregnancy have also been described in Sweden and other European countries (12, 45). Pregnant Norwegian women are advised to use supplementation with 400 mg of folic acid/day, starting 1 month before conception until 2–3 months of pregnancy (46), as food is not fortified with folic acid. More than half of our study population reported a daily fruit consumption below the RI. It is likely that an inadequate intake of fruit contributes to low folate intakes in many of the pregnant women. Although the prevalence of iodine deficiency among pregnant women in Europe has been well-known for decades, intake still seems to be insufficient in many countries, including Norway (47). A quarter of the women in our study reported iodine intakes below the RI, in line with a representative Norwegian population (Norkost 3), showing unsatisfactory iodine intakes in 46% of women from the general population (48). Other studies have revealed mild-to-moderate iodine deficiencies in a large number of pregnant Norwegian women (49), likely because diet alone seems to be insufficient in maintaining adequate iodine concentrations (50). Iodine deficiency can cause severe adverse effects in the child, such as impaired cognitive outcomes and delayed neurodevelopment (51, 52). Reported vitamin D intake was higher in our study (13.6 μg/day) compared to a large Norwegian cohort study of more than 40,108 pregnant women conducted approximately 15 years ago, in which 63% did not reach the RI level of 10 μg/day, in spite of high vitamin D supplementation rates (19). Public health efforts in the past decade might have increased the awareness of Vitamin D and its importance, and contributed to higher intake rates in our study. Although previous studies have generally reported sufficient maternal calcium intakes in the United States, the United Kingdom, and Europe, we found a significant number of women at risk for potentially inadequate intakes (12). Low selenium intakes have also previously been reported in Western countries, particularly in inhabitants of Northern Europe (53). In a study of 230 pregnant women in the United Kingdom, the majority had an overall low selenium status (54). Low maternal selenium levels in pregnancy may adversely affect the child’s psychomotor development (55, 56). Adequate maternal zinc intake is also crucial for normal fetal development (57). Most women in our study reported zinc intakes above the RI, which is in agreement with previous reports from other European countries (12).

### Coffee and alcohol

Daily caffeine intake was higher in our study than in the MoBa study, which assessed caffeine intake in 59,123 pregnant women in Norway and found that 10% of the women exceeded the caffeine level of 200 mg/day (41). The high rate of women exceeding recommendations for coffee intake in our study (41%) is a matter of concern. Maternal caffeine intake in pregnancy has been associated with reduced birthweight in relation to gestational weight in the Norwegian MoBa study, a large prospective observational cohort study (58), in line with other studies (59–61). Although women in our study tended to give up lifestyle choices that could harm their unborn child, such as the consumption of nicotine-containing products (62), we found a high number of women reporting alcohol intake in the first 4–5 months of pregnancy. The adverse effects of alcohol on the fetus are well known and an important reason for many women to abstain from alcohol during pregnancy (63). Severe effects on the fetus are seen with an alcohol intake above 24–48 g/day; however, even lower levels of alcohol consumption can adversely affect the developing fetus. Due to uncertainty of the threshold level for negative health effects in the offspring, it is recommended to totally abstain from alcohol in pregnancy (64). A large meta-analysis concluded that approximately 10% of women globally report alcohol intake in pregnancy. The highest prevalence was seen in European countries, where up to a quarter of women consumed alcohol in pregnancy (15). The large Norwegian MoBa study reported alcohol consumption among 31.8% of women in the first trimester and among 9.7% in the second trimester. The majority of these women consumed alcoholic beverages less than once a month (65). Contrary to these findings, a recent study comprising 575 Norwegian women found that 27% reported weekly alcohol intake before pregnancy, compared with none in early pregnancy (66). We found a higher alcohol consumption in women with higher educational level, which is comparable to the findings of the MoBa study (65). Similarly, a Danish study in 100,000 pregnant women found that binge drinking in the pre-recognized part of pregnancy was more common among well-educated women (67). It has been suggested that alcohol consumption in pregnancy might increase around the globe in the coming years, which could cause a surge of alcohol-related birth defects (68). Our findings therefore warrant special attention.

Apart from a higher intake of alcohol and coffee, our results suggest that women with a higher educational level eat more healthy foods, such as whole-grain foods, vegetables, foods rich in omega 3, and have a reduced intake of red meat. The tendency toward better dietary quality in these women was not surprising, as higher education independently influences better food choice (69). A large body of evidence points to the influence of social class on diet and lifestyle (70, 71). Similar to our findings, a Spanish study assessing dietary intake and compliance to national food guidelines found that less educated women reported lower intakes of omega-3 fatty acids, and higher intakes of saturated fatty acids (72). Greater financial and social resources possibly provide for better means to support a healthy lifestyle, although we found no association between income and dietary intake in our study (70).

## Strengths and limitations

A major strength of the present study is the robust sample size from a general population, which ensures a large variation of dietary habits. All FFQs are subject to large between-person errors. In addition, self-reported data are prone to systematic bias and methodological challenges (73), such as social desirability bias. This bias regularly occurs because study participants tend to answer questions in a way that will be viewed favorably by others, which leads to overreporting of good dietary habits and underreporting of unhealthy habits. Consequently, overreporting intakes of fiber and healthy food items, such as vegetables and fruit, is a common issue with FFQs. In spite of its limitations, the FFQ remains the most-used instrument, especially in large cohorts.

Women in the PreventADALL study are older, wealthier, better educated, and less often smokers and single, compared with the general pregnant population in Norway, which might limit the generalizability of our results (74, 75). Moreover, our dietary intake data may not be representable for pregnant women in other countries or other ethnicities, and therefore the implications may differ in other settings.

## Conclusion

Dietary intake in a large cohort of Norwegian pregnant women in a mother–child birth cohort showed low adherence to recommendations with regard to saturated fat, total carbohydrates, folate, iron, calcium, vitamin D, and iodine. Women with a university degree showed a tendency towards healthier eating habits, except for higher intakes of alcohol and coffee. Thus, our results highlight the role of education in dietary decision-making.

A sufficient nutrient supply in pregnancy is crucial for ensuring favorable maternal and fetal health outcomes. Intervention strategies, aimed at educating pregnant women and encouraging healthful dietary choices, may therefore be needed for women at all educational levels.

## Supplementary Material

Food and nutrient intake and adherence to dietary recommendations during pregnancy: a Nordic mother–child population-based cohort

## Clinical trial registration

The ClinicalTrials.gov registration number for this study is NCT02449850.
