# Supplementary material for: Food and nutrient intake and adherence to dietary recommendations during pregnancy: a Nordic mother–child population-based cohort
Source: Food Nutr Res. 2019 Dec 20;63:10.29219/fnr.v63.3676. doi: 10.29219/fnr.v63.3676 (PMC6939665; doi:10.29219/fnr.v63.3676)
Supplement: Food and nutrient intake and adherence to dietary recommendations during pregnancy: a Nordic mother–child population-based cohort [file FNR-63-3676-s001.pdf]

1 # Supplementary tables and figures

1. Supplementary table, General background characteristics

**Anthropometric characteristics in present cohort** *(N=1674)*<br>*Height measured at inclusion<br>**Pre-pregnancy				**PreventADALL study cohort**<br>*(N 2701)*
	**Mean (SD)**	**Min**	**Max**	**Mean (SD)**
**Age mother** (years)	32.5 (4.1)	21.0	48.0	32.3 (4.2)
**Height mother** (cm)*	168.2 (6.3)	147.0	187.0	168.0 (6.2)
**Weight mother** (kg)**	65.5 (11.1)	42.0	124.0	65.5 (11.4)
**BMI ** **	24.6 (3.5)	17.2	41.4	24.9 (3.8)
	**Categories**	**N**	**%**	*(N 2349) %*
**Mother Nordic origin** *(N 1520)*		1379	90.8	90.1
**Marital status**	*Married*	604	39.7	41.2
(N=1520)	*Cohabitants*	872	57.4	55.9
	Single	26	1.7	1.9
	Other	18	1.2	1.0
**Maternal education level**	*Preliminary school only (9/10y)*	9	0.6	0.8
(N=1513)	*High school only*	100	6.6	10.2
	Higher education <4y	478	31.6	32.4
	Higher education 4 y or more	882	58.3	53.7
	PhD	43	2.8	2.9
	Other education	1	0.1	0.1
**Paternal education level**	*Preliminary school only (9/10y)*	16	1.1	1.3
(N=1470)	*High school only*	232	15.8	19.4
	Higher education <4y	453	30.8	30.0
	Higher education 4 y or more	700	47.6	44.7
	PhD	54	3.7	3.4
	Other education	2	0.1	0.3
	None of the above	13	0.9	0.9
**Maternal Work**	*Fulltime*	1299	81.0	73.3
(N=1604)	*Part-time*	129	8.0	8.0
	Student	103	6.4	5.5
	Housewife/ homemaker	16	1.0	0.9
	Job-seeker/unemployed	22	1.4	1.2
	Disabled	10	0.6	0.3
	Other	25	1.6	1.4
**Gross income household (NOK)**	*Below 300 000*	15	1.0	1.3
(N=1520)	*300 000 -600 000*	142	9.3	13.0
	600 000- 1 000 000	562	37.0	40.8
	1 000 000 – 1 400 000	572	37.8	31.6
	> 1 400 000	210	13.8	11.5
	Did not want to answer	17	1.1	1.7
**Previous pregnancies** (*N1520)*	*Yes*	810	53.3	55.0
**Previous deliveries**	*0*	246	30.4	27.6
(N=810)	*1*	455	56.2	57.4
	2	96	11.9	13.2
	3	8	1.0	1.3
	4	3	0.2	0.4
	6	1	0.1	0.1
	8 or more	1	0.1	0.1
**Living environment**	*City, densely populated*	678	44.6	39.0
(N=1520)	*City, less densely populated*	608	40.0	37.5
	Suburb	113	7.4	15.9
	Village	90	5.9	2.2
	Countryside, outside village	31	2.0	5.4
**Tobacco use** (previous and/or current)	*Ever*	455	29.9	27.9
(N=1520)	*Smoking*	362	23.8	

	Snus	369	24.3	22.5

2. Supplementary table, Dietary intake fat (N=1674)

Variable	*Unit*	Median	IQR	Min	Max	5th	95th
**Fat**	*g/day*	**92.4**	45.6	29.1	232.8	50.5	161.7
**Saturated**	*g/day*	**33.5**	18.0	7.9	95.3	17.7	61.0
**Trans**	*g/day*	**0.77**	0.48	0.06	4.04	0.32	1.66
**Monounsaturated**	*g/day*	**43.3**	17.8	10.4	98.4	18.4	60.9
**Polyunsaturated**	*g/day*	**15.7**	9.0	3.9	71.3	8.0	31.1
**Cholesterol**	*g/day*	**307**	162	67	1218	155	585
**Omega 3**	*g/day*	**3.9**	2.6	0.6	15.8	1.8	8.1
**Omega 6**	*g/day*	**11.8**	7.1	3.5	54.8	6.0	24.1

3. Supplementary table, Daily dietary intake of vitamins and nutrients *(N=1674)*

Variable	*Unit*	Median	IQR	5th Perc	95th Perc	***RI*** *(NNR)*	***% of women adhering to NNR***
**Vitamin A**	*RE* [a]	**1694**	1101	661	3313	**800**	**90.4**
**Vitamin C**	*mg*	**207**	136	86	425	**85**	**95.6**
**Vitamin D**	*µg*	**13.6**	12.1	4.2	32.6	**10**	**71.3**
**Vitamin B 6**	*mg*	**2.7**	2.3	1.3	6.6	**1.4**	**93.4**
**Vitamin B 12**	*µg*	**7.9**	4.1	4.0	14.5	**2.0**	**99.7**
**Folate**	*µg*	**480**	275	236	921	**500**	**45.6**
**Tocopherol**	*α-TE*	**25.1**	17.2	10.2	48.8	**10**	**95.3**
**Thiamine**	*mg*	**2.36**	1.78	1.1	5.2	**1.5**	**83.2**
**Riboflavin**	*mg*	**2.8**	2.2	1.3	6.2	**1.6**	**86.7**
**Niacin**	*NE* [d]	**30.0**	23.1	14.2	65.7	**17.0**	**88.8**
**Iodine**	*µg*	**256**	189	103	563	**175**	**75.6**
**Magnesium**	*mg*	**463**	206	260	770	**280**	**92.4**
**Zinc**	*mg* [c]	**15.5**	14.8	7.7	43.4	**9**	**89.8**
**Calcium**	*mg*	**1045**	558	495	1960	**900**	**63.8**
**Potassium**	*g*	**4.73**	2.10	2.68	7.75	**3.1**	**89.9**
**Selenium**	*µg*	**69**	52	31	162	**60**	**58.7**
**Copper**	*mg*	**1.83**	1.94	0.87	5.60	**1.0**	**90.0**
**Phosphorus**	*mg*	**1876**	820	1060	3108	**700**	**99.9**
**Iron**	*mg* [b]	**15.1**	10.0	7.3	92.0	*	*
**Manganese**	*mg*	**0.0**	2.5	0.0	5.0	**	**

IQR: Interquartile range, RI: Recommended intake (NNR2012) in pregnancy

[a] Retinol equivalents; 1 retinol equivalent (re) = 1 µg retinol = 12 µg β-carotene.
α-tocopherol equivalents; 1 α-tocopherol equivalent (α-te) = 1 mg rrr α-tocopherol
[b] Meal composition influences the utilization of dietary iron. Availability increases if the diet contains abundant amounts of vitamin C and meat or fish daily, and it is decreased with simultaneous intake of polyphenols or phytic acid.
[c] The utilization of zinc is negatively influenced by phytic acid and positively influenced by animal protein. The recommended intakes are valid for a mixed animal/vegetable diet. For vegetarian cereal-based diets, a 25%–30% higher intake is recommended
[d] Niacin equivalent; 1 niacin equivalent (ne) = 1 mg niacin = 60 mg tryptophan
*Iron balance during pregnancy requires iron stores of approximately 500 mg at the start of pregnancy. The physiological need of some women for iron cannot be satisfied during the last two thirds of pregnancy with food only, and supplemental iron is needed.
** Too limited data to determine requirements during pregnancy

4. Supplementary table, Intake of foods in g/day

Variable	Median	IQR	5th Perc	95th Perc
**Bread**	**140**	114	35	311
**Cereals**	**130**	116	34	336
**Cake**	**20**	24	3	63
**Potatoes**	**36**	43	5	117
**Vegetables**	**363**	261	141	802
**Fruit, berries**	**430**	327	143	1008
Fresh	**221**	188	61	623
Jam, conserves	**12**	20	0.2	52
Juice, smoothie	**144**	240	6	501
Nuts, olives, seeds	**7**	15	0	50
**Meat**	**112**	78	28	225
Red meat	**74**	55	17	157
Poultry	**33**	39	2	97
**Fish and seafood**	**77**	64	17	182
Low-fat, half-fat	**12**	19	0	44
Fat	**20**	19	1	58
Fish Products	**1**	4	0	13
unspecified	**6**	12	0	30
Shellfish, entrails	**2**	4	0	14
Fish spread	**4**	15	0	43
Sushi	**14**	28	0	67
**Egg**	**20**	21	3	64
**Dairy products**	**345**	326	53	920
Milk, yoghurt	**316**	318	35	881
Full cream milk	**0**	2	0	143
Semi-skim milk	**10**	58	0	308
Skim milk	**0**	2	0	300
Milk unspecified	**8**	11	0	25
Yoghurt	**62**	97	0	301
Milk flavored	**5**	29	0	128
Cultured milk	**0**	2	0	57
Quark	**0**	9	0	49
(Ice-, sour-) cream	**25**	30	4	92
**Cheese**	**29**	24	8	75
**Butter, margarine**	**27**	29	5	77
Margarine	**1**	1	0	3
Low-fat margarine	**0**	11	0	40
Butter	**1**	13	0	41
unspecified	**3**	3	1	11
Oil, other fat	**1**	1	0	3
Mayonnaise, dressing	**4**	10	0	28
**Sugar, sweets**	**19**	23	3	68
**Beverages**	**2061**	1019	936	3646
Coffee	**203**	385	0	878
Tea	**92**	231	0	768
Juice, soft-drink	**19**	18	0	291
Juice, soft-drink (sugar-free)	**19**	143	0	701
Wine, beer (alcohol-free)	**5**	36	0	143
Beer	**0**	5	0	143
Wine, Liquor	**0**	3	0	64
Wine	**0**	2	0	63
Liquor	**0**	0	0	1
Drinks	**0**	0	0	7
